# Supplementary material for: A New Lignan from Annona squamosa L. (Annonaceae) Demonstrates Vasorelaxant Effects In Vitro
Source: Molecules. 2023 May 23;28(11):4256. doi: 10.3390/molecules28114256 (PMC10254816; doi:10.3390/molecules28114256)

Figure S1 HR-ESI espectra of esquamosan

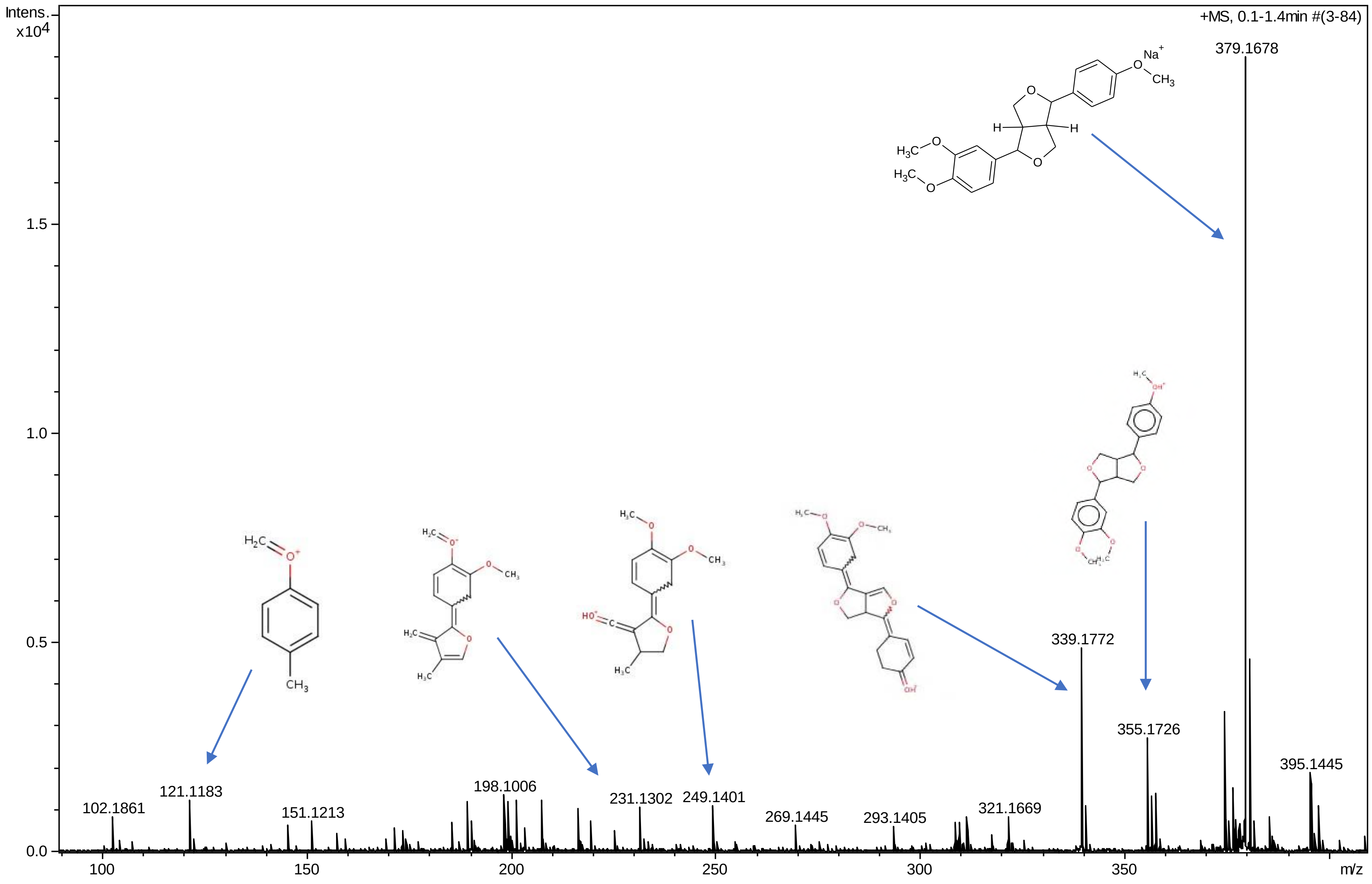

Figure S2 ^{1}H NMR espectrum of esquamosan

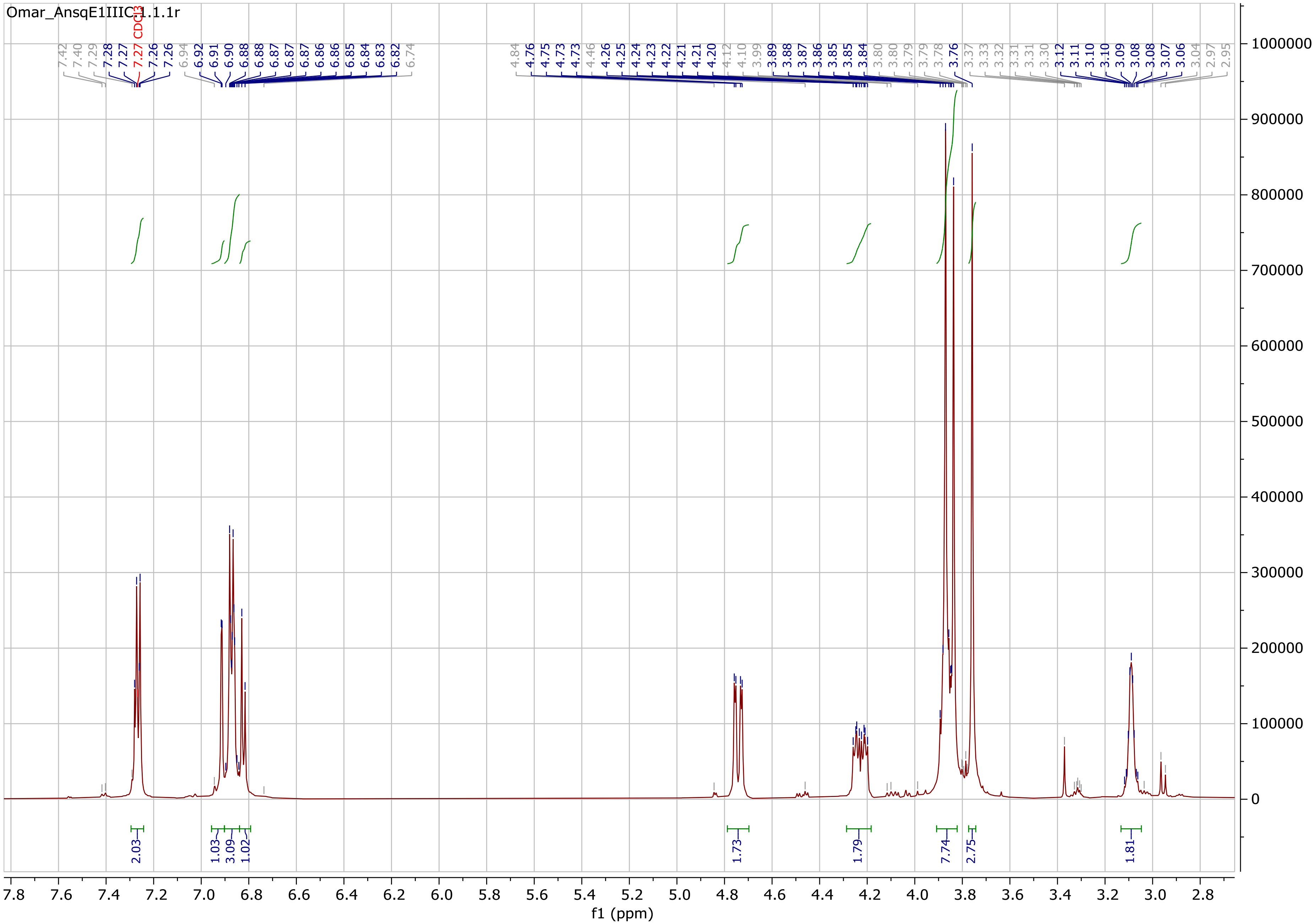

Figure S3 13C NMR and DEPT 135 and ^{13}C NMR espectrum of esquamosan

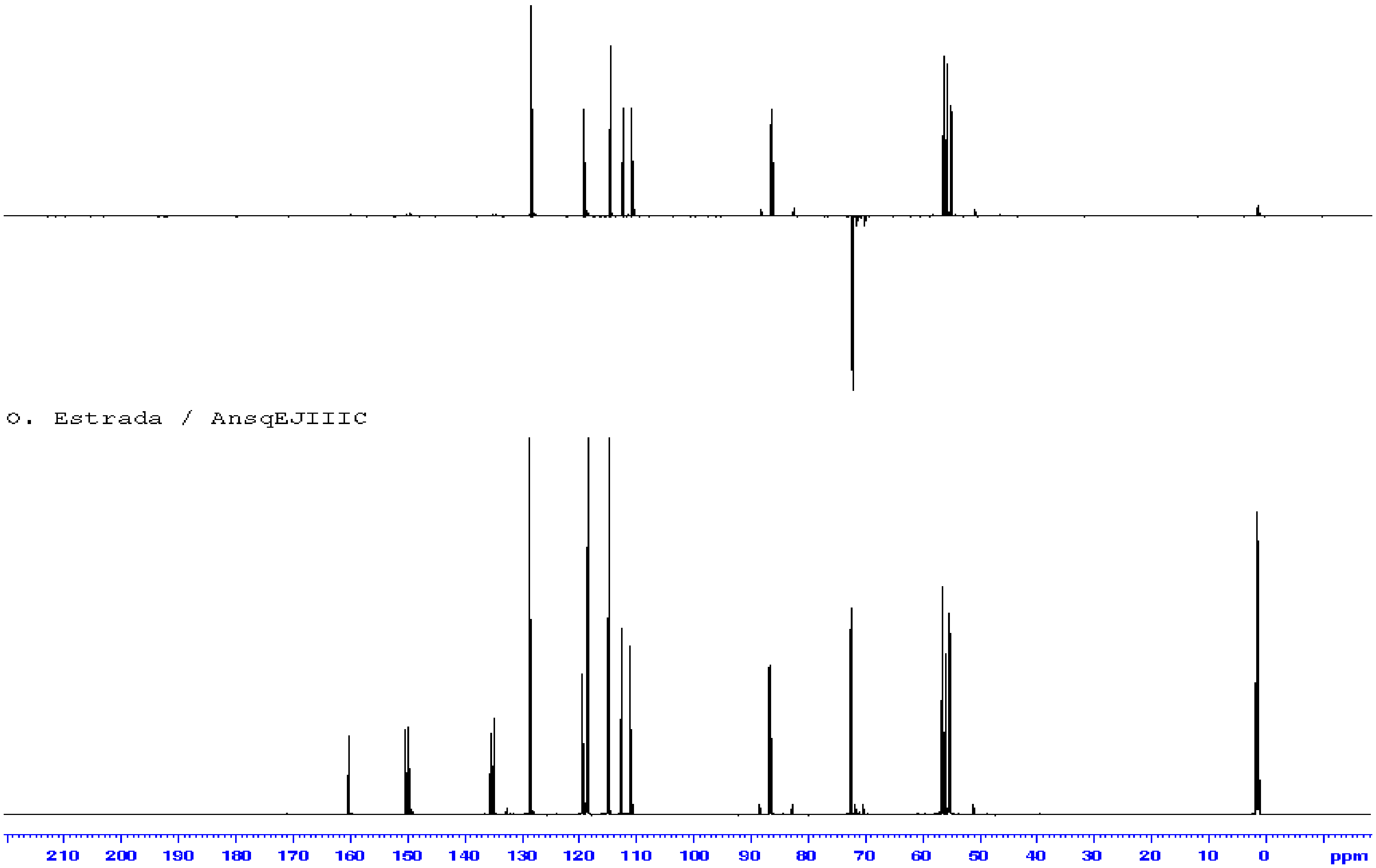

Figure S4 HSQC espectrum of esquamosan

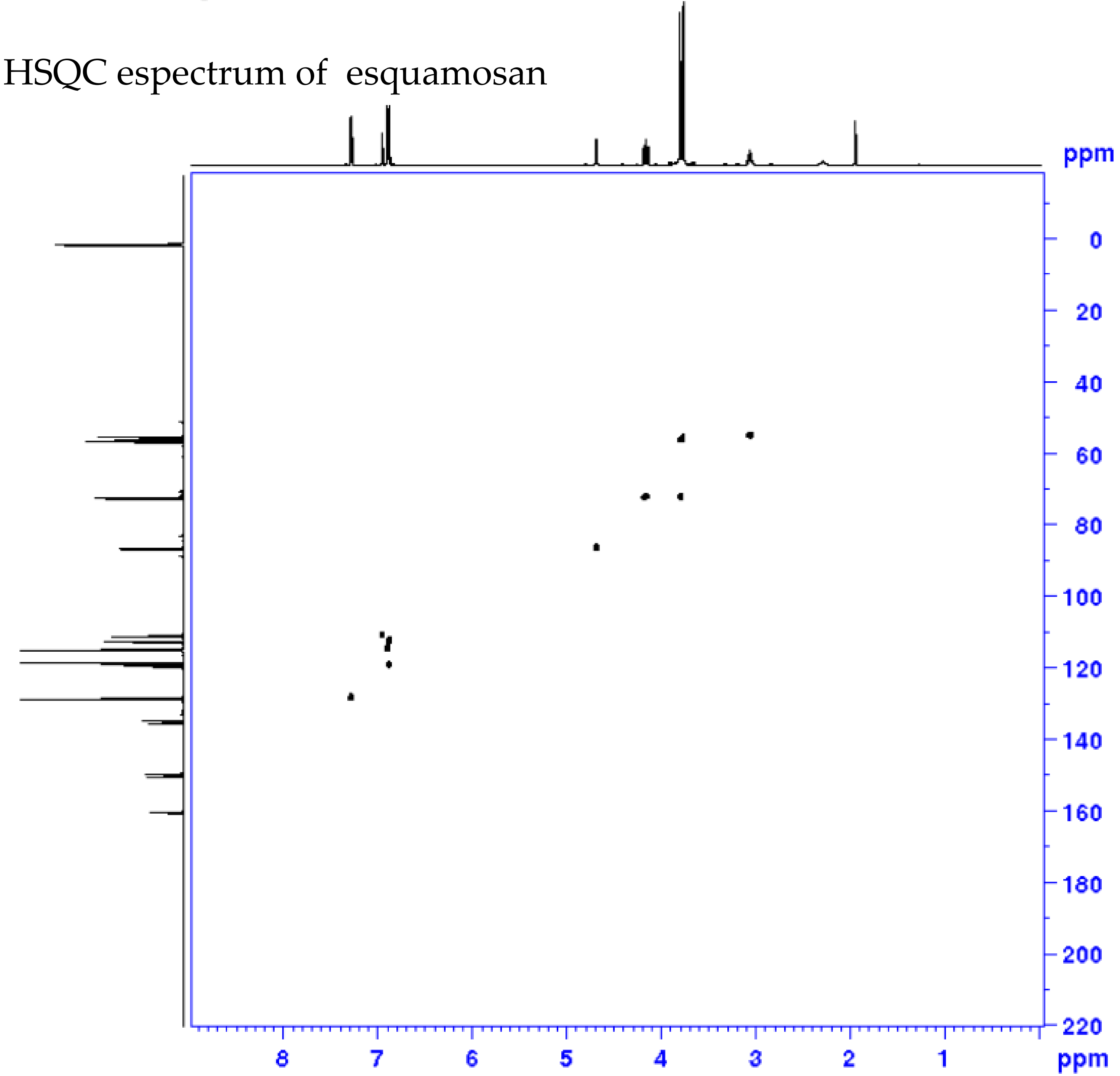

Figure S5 HMBC espectrum of esquamosan

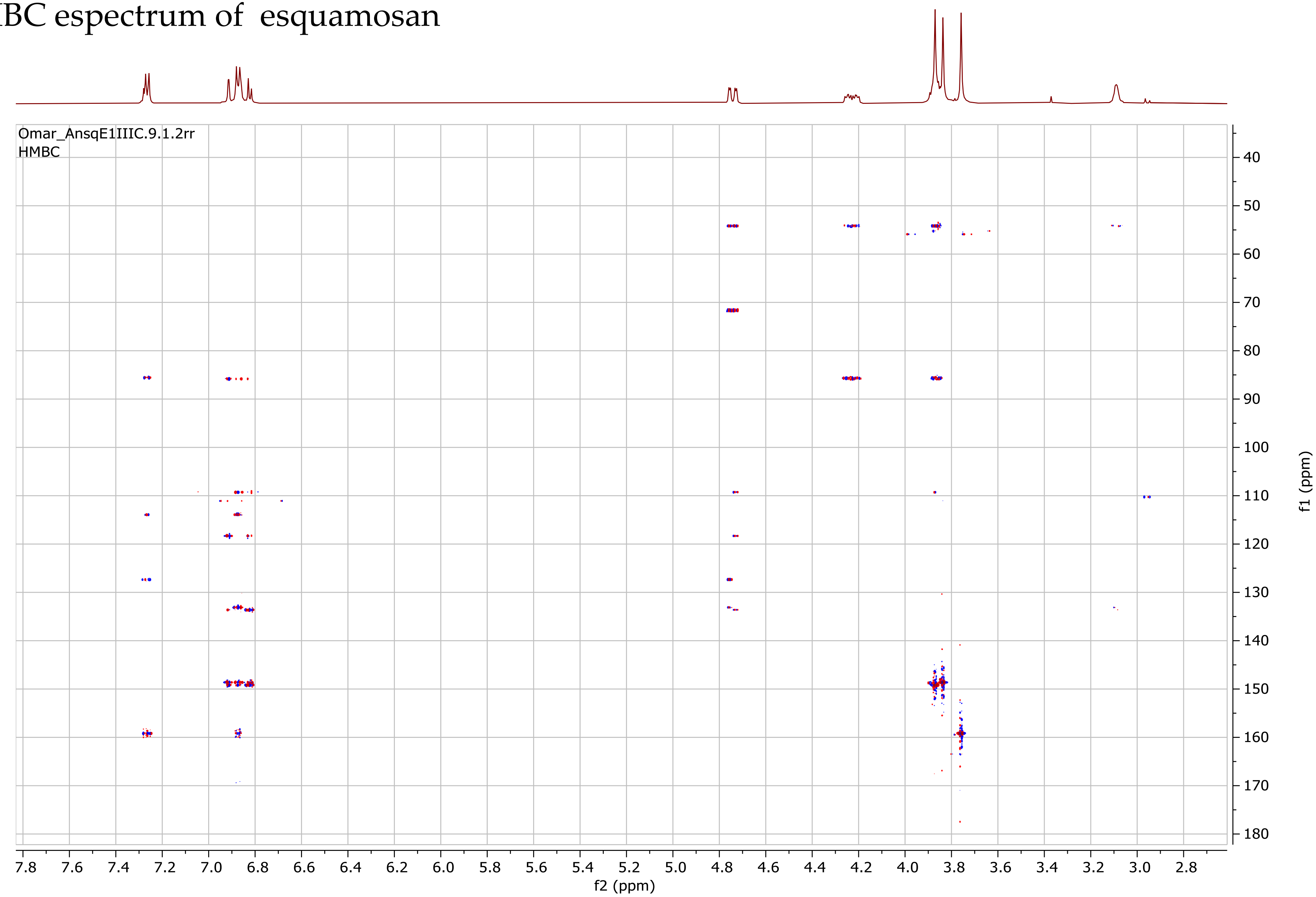

Figure S6 NOESY espectrum of esquamosan

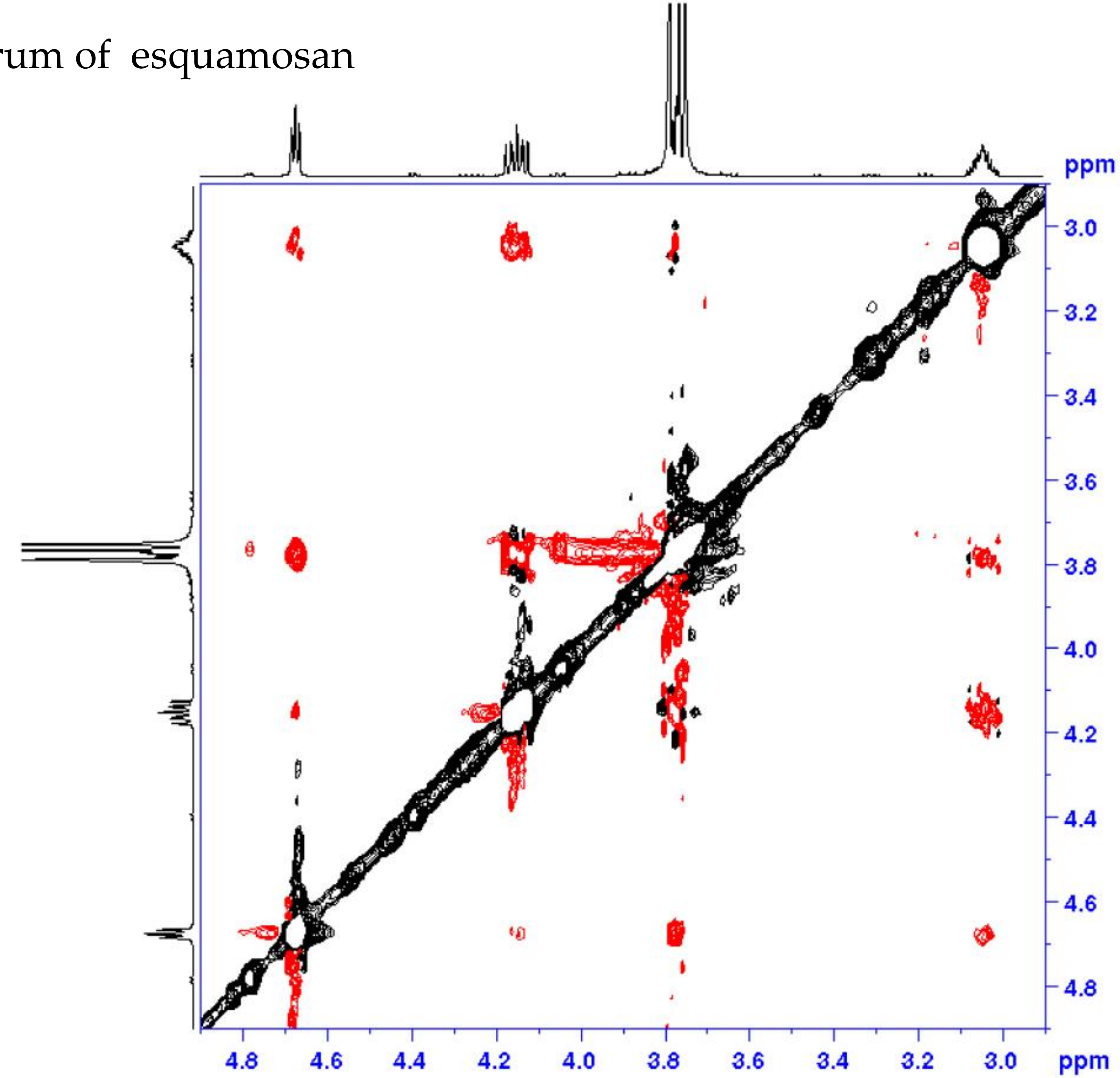

Figure S7 COSY espectrum of esquamosan

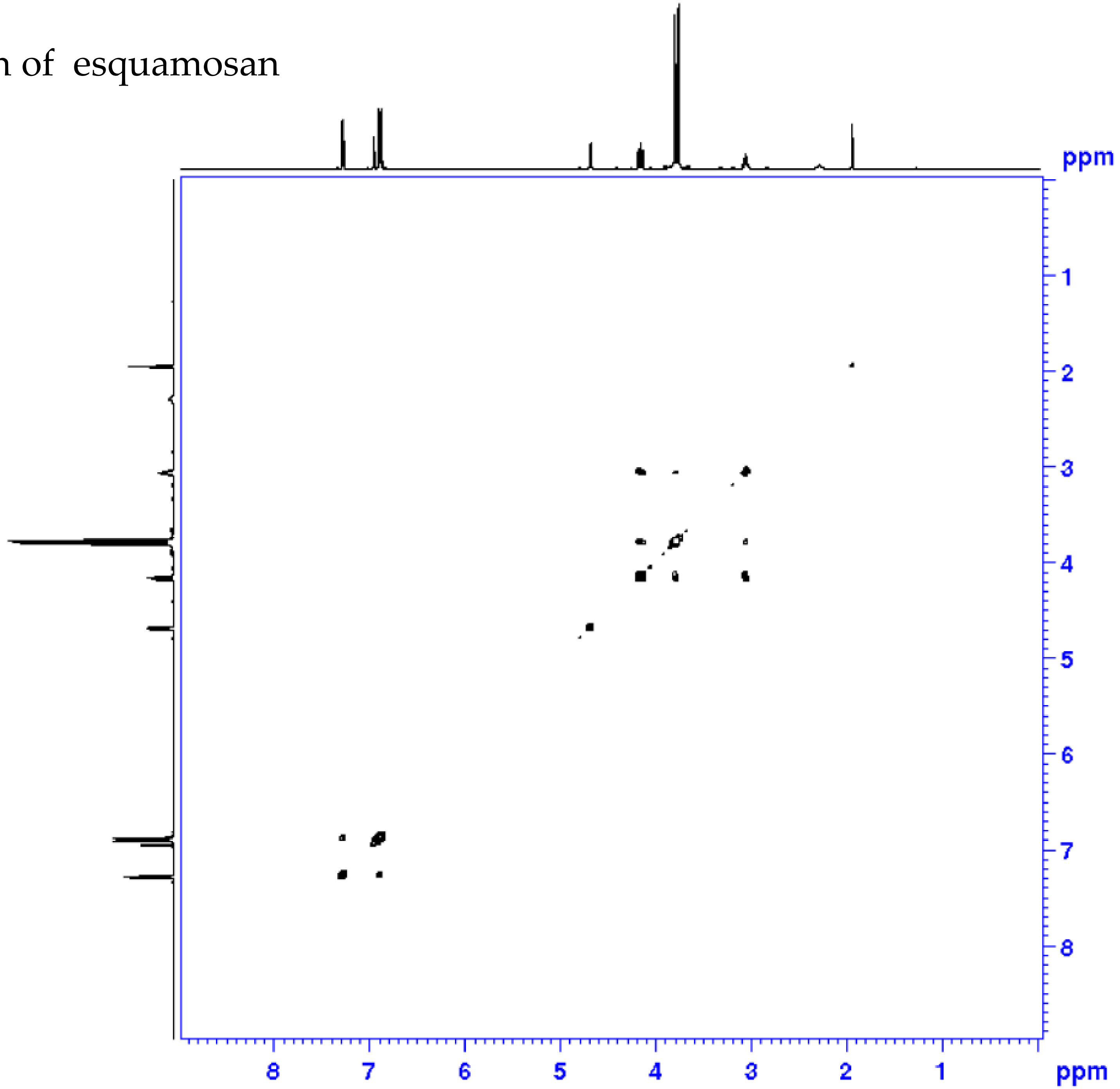

Supplement: Supplementary file 1 [file molecules-28-04256-s001.zip › molecules-2386105-supplementary.pdf]
